# Reconstruction of Surgical Defects of the Oral Cavity with Bilayer Dermal Matrix: Our Experience

**DOI:** 10.3390/jcm14238534

**Published:** 2025-12-01

**Authors:** Andrea Ferri, Mara David, Giulia Salti, Giovanni Lilloni, Bernardo Bianchi, Silvano Ferrari

**Affiliations:** 1 Department of Maxillofacial Surgery, University Hospital of Parma, 43126 Parma, Italy; a.ferri@libero.it (A.F.); giuliasalti@libero.it (G.S.); giovanni.lilloni.md@gmail.com (G.L.);; 2 Department of Maxillofacial Surgery, San Martino Hospital, 16132 Genova, Italy

**Keywords:** acellular dermal matrix, reconstruction, oral cancer, squamous cell carcinoma

## Abstract

**Purpose:** Reconstructive options for mucosal defects of the oral cavity resulting from the resection of tumors include primary closure, mucosal and split thickness skin grafts, pedicle flaps, and microvascular free flaps. Lately the use of an acellular dermal bilayer matrix has been introduced for the reconstruction of superficial mucosal defects of the oral cavity. **Methods:** Twenty-one patients treated for SCC of the oral cavity with intraoral resection and simultaneous reconstruction using a bilayer dermal matrix between 1 January 2020 and 31 December 2024 with at least 6 months of follow-up were retrospectively considered. Data were collected regarding the site of the lesion, the initial TNM staging, the size of the surgical defect, the timing of silicone sheet removal, the complications and the long-term outcomes. **Results:** Tumor site included the tongue in 16 cases, the hard palate in 1 case, the cheek in 2 cases, the floor of the mouth in 1 patient, and the inferior lip in 1 patient. Re-epithelialization was achieved in all cases within 21 days. No major complication was observed. **Conclusions:** Bilayer dermal matrix demonstrated to be an excellent option for small and superficial oral cavity reconstruction if proper indications are followed.

## 1. Introduction

Oral cancer represents the sixth most common cancer in the world.

Squamous cell carcinoma (SCC) is the most common histology, and the main etiological factors are tobacco and alcohol use. Worldwide, 405,000 new cases of oral cancer are anticipated each year, and the annual mortality is about 145,000 deaths. High incidences of oral cancer are found in South and Southeast Asia, areas of the West and Eastern Europe, Latin America and the Caribbean and Pacific regions [1].

Oral cancer is more common in men and usually occurs after the fifth decade of life. The main treatment, when possible, is resective surgery. Reconstructive options for mucosal defects of the oral cavity resulting from the resection of tumors include primary closure, mucosal and split thickness skin grafts, pedicle flaps, and microvascular free flaps. In recent years, the use of acellular dermal bilayer matrix has been introduced for the reconstruction of superficial mucosal defects of the oral cavity, with results supported by several studies in the literature confirming the safety and efficacy of these devices [2]. In terms of indications, dermal matrix is a candidate for the replacement of skin grafts, which have been used for years in the management of these defects, and as an alternative to local flaps, particularly for more superficial defects [3]. The dual-layer design, with a silicone sheet temporarily protecting the matrix from the action of saliva, is the main biological advantage of these devices [4], while the absence of donor sites, ease of use, and prevention of bleeding and pain provided by the protection of the surgical site are the main benefits to the patient [5]. Despite its wide use, there are still some gaps in the international literature, mainly concerning the absence of large case series, ideal definition of the timing of silicone sheet removal, and analysis of complications and functional outcomes. Only a few papers have been published in recent years, and many of them are focused only on technical or descriptive aspects, without a critical analysis of indications and long-term results. Moreover, the lack of multicenter prospective trials makes the evaluation of this device difficult to assess. The purpose of this study was to further analyze these aspects with the support of a clinical case series.

## 2. Materials and Methods

The investigations for the following study were conducted in accordance with the principles outlined in the Declaration of Helsinki and were approved by the Ethics Committee of the University of Parma on 15 May 2025, protocol number 15465, code 675/2024/OSS/AOUPR ID SIRER 6850 INTEGRA 2023. Inclusion criteria were treatment for squamous cell carcinoma of the oral cavity with intraoral resection and simultaneous reconstruction using bilayer dermal matrix between 1 January 2020 and 31 December 2024 with at least 6 months of follow-up, and the release of a consent form. Data extrapolated from medical records were retrospectively considered and are listed in Table 1. Informed consent to participate in the study was obtained from all enrolled patients.

**Figure 1:**
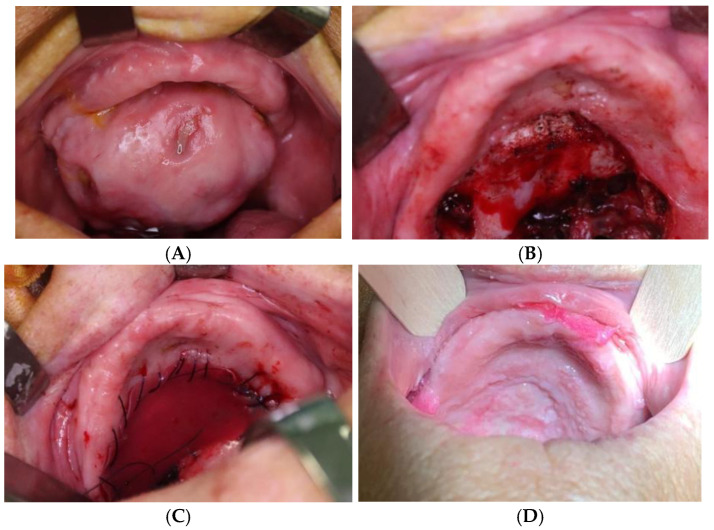
(**A**) Huge pleomorphic adenoma of hard palate; (**B**) bone-sparing surgical removal; (**C**) reconstruction with bilayer dermal matrix; (**D**) 1-month follow-up result.

**Figure 2:**
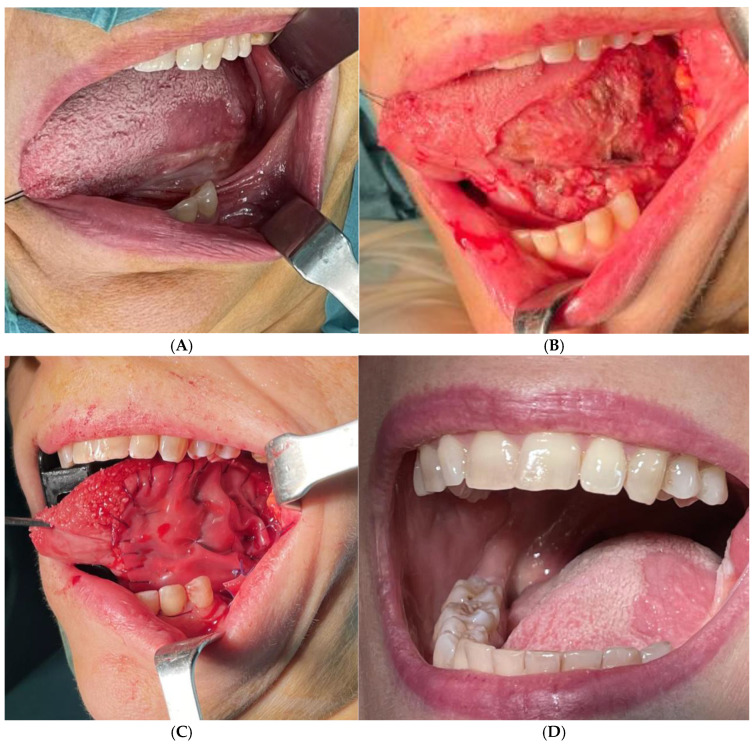
(**A**) Squamous cell carcinoma in situ of the left lateral border of the tongue; (**B**) superficial mucosectomy of the left lateral border; (**C**) application of bilayer dermal matrix; (**D**) 6-month follow-up result.

For each patient, data were collected regarding the site of the lesion, the initial TNM staging, the size of the surgical defect, the timing of silicone sheet removal, the complications, and the long-term outcomes. In all patients, a surgical resection of the tumor was performed (Figure 1 and Figure 2). The reconstruction was achieved simultaneously by applying a dermal bilayer matrix that was carefully shaped to cover the defect (Figure 1 and Figure 2). All the resective procedures were performed using an intraoral approach, and in all patients, after the tumor resection, the dermal matrix was fixed with a continuous sealing resorbable suture. Central stitches were placed to maintain silicone adherence to the underlying surface. Functional and quality-of-life outcomes were assessed through administration of the validated University of Washington Quality of Life Questionnaire (UW-QOL) at the 6-month follow-up [6]. Data extrapolated from the questionnaires are listed in Table 2.

Patients were evaluated at the preoperative time and at postoperative weeks 1 and 3 and 6 months later to analyze silicone sheet removal timing, healing process of the intraoral wounds, and local complications (Figure 1 and Figure 2).

## 3. Results

A total of 21 patients were enrolled in the study: 8 were male, and 13 were female. Tumor sites included the tongue in 16 cases, the hard palate in 1 case, the cheek in 2 cases, the floor of the mouth in 1 patient, and the inferior lip in 1 patient. Tumors were clinically staged as cTis in 3 patients, cT1 in 12 cases, and cT2 in 6 cases. In total, 20 patients were clinically N0M0, only 1 patient was clinically N2a. In all cases, histology confirmed TNM clinical staging, so the pathologic TNM were pTis in 3 cases, pT1 in 12 cases, and pT2 in 6 cases. In 20 patients, the histological diagnosis was SCC, while in only 1 case, it was pleomorphic adenoma. The most affected site of the oral cavity according to the pathology was the tongue, with 16 cases.

The average size of the surgical defect obtained with surgical resection with acellular dermal matrix was 3.5 × 4.2 cm. A nasogastric tube was placed in all patients and maintained for a time ranging from 3 to 5 days to prevent wound dehiscence.

Neck was treated according to the N0 staging with a wait-and-see approach in 6 cases, sentinel node biopsy in 6 cases, and elective neck dissection (staged 1–3 levels) in the remaining 9 patients.

The mean hospitalization time was 5 days, and all patients were discharged on a soft diet. The silicone sheet was planned to be maintained in place for a period of 21 days, but the actual removal timing ranged from 13 to 21 days, with earlier removal related to unwanted silicone displacement, while delayed removal was due to a delay in patient return for personal reasons. Re-epithelialization was achieved in all cases within 21 days, independent of the dehiscence of the silicone sheet.

No major complications were observed. Minor bleeding was observed in 2 patients and controlled with bipolar cauterization. No cases of infection were recorded.

The subjective “University of Washington Quality of Life” test was administered to all subjects at the 6-month follow-up. Values are expressed as a percentage from 0 to 100, where 0 is the worst achievable result, and 100 is the best result. All results are summarized in Table 2. All patients reported no pain and bleeding during the postoperative period or after discharge from the hospital, as pointed out by questionnaire values, with 18 patients not experiencing severe postoperative pain, 19 patients not experiencing severe speech problems, and 18 not experiencing chewing problems.

In contrast, it emerged that 3 experienced pain, 2 experienced difficulty in chewing, and 3 experienced difficulty in lingual motility during the first postoperative week.

## 4. Discussion

Management of small (Tis-T1-T2) squamous cell carcinoma of the oral cavity is today well defined, being resection with free margins the mainstay of treatment. Some debate still exists in the literature about the ideal treatment for the N0 neck, with follow-up, sentinel node biopsy, or elective neck dissection the most commonly used approach [7]. Independent of the different oncological management of these patients, which is not the topic of the present paper, when a small, superficial defect involving the oral mucosa has to be reconstructed, different options are available for the surgeon.

Direct suture is the simplest one, but in many cases it could lead to distortion or anchorage of the involved structures, with subsequent unsatisfactory results in terms of form and function: in the case of the tongue, limitations of movement could be related to impairment of swallowing and speaking; in the case of the cheek, limitations of mouth opening and oral commissure distortion; in the case of the palate, it is usually not feasible at all.

Secondary healing is certainly an option for such small defects, and often final results are satisfactory, but this means a very unpleasant postoperative recovery for the patient, with pain, bleeding, and infection often observed as complications during the first postoperative weeks.

Skin grafts have been used for a long time in the past but have been recently abandoned by many surgeons because healing is often unpredictable, a donor site is required, and dressings that could increase the success rates of the procedure are very uncomfortable for patients (packages or sponges).

The ideal reconstruction should indeed consist of the replacement of a tissue with the same features as the resected one, and the paradigm of like-for-like should always be considered a guide in reconstructive planning. In this context, local flaps are surely a great option and are widely used for many oral defects resulting from T1 and T2 tumor resections [8]. In particular, buccinator miomucosal flaps (in all their technical variants) are a great option in many of these defects, and in our center, they are routinely used in many cases [9]. However, buccinator flaps require experience, and despite a very low associated morbidity, are related to violation of a local donor site [10]. Furthermore, in many cases, a second surgical step for pedicle resection is planned, with temporary patient discomfort and delay in the return to normal life. Finally in the case of superficial defects, the thickness of the flap could be even too much, with an excess of volume that could limit final results.

The use of a dermal matrix for intraoral reconstruction is not new, and its reliability and efficacy have already been documented in the literature [11]. However, only a few studies have been presented, and most do not analyze some critical issues that are usually encountered in clinical practice, such as the ideal indications for the technique, advantages of the bilayer matrix with the correct management of the silicone sheet during the healing process, and versatility of the device in different subsites of the oral cavity.

Ideal indications for the dermal matrix are superficial defects, when only a few millimeters of lining have to be restored, and when the receiving bed is well vascularized. In particular, in the case of tongue defects, the dermal matrix is the ideal solution after a mucosectomy, or in the case of defects involving the lateral border, when only the lining has to be reconstructed [12,13]. In the case of floor of the mouth involvement, a buccinator flap should be preferred to prevent anchorage of the tongue to the mouth floor. Extension in length instead does not represent a real limit, being the only limitation related to the length of the membrane available. When a tongue defect is approached, the key point of the procedure is the correct fixation of the matrix on the receiving bed: extreme care must be taken in the complete sealing of matrix border, and a continuous suture represents, in our experience, the best option. In addition, the use of some central stitches that fixed the silicone sheet to the bed is essential to prevent the “tent” effect that would bring about early silicone detachment. All these precautions are essential in a mobile structure, such as the tongue, when packages and compressive dressings are not feasible. In the series presented here, this approach led to very satisfactory results with restoration of a normal lining in a few weeks. We also combined a direct suture with the matrix in 2 cases; we chose this combination when direct sutures alone led to a distortion of the tongue, especially when the tip was involved. In such cases, the tip was sutured, and the border was reconstructed with the matrix. As already said, the main indication for the tongue is superficial defects; we also used the membrane as a second choice option in thicker defects observed in 5 cases. It could be performed only in cases of floor of the mouth preservation and only in patients with a poor general status, when buccinator flaps were not indicated. In such cases, although the shape of the tongue was not fully restored, the function of the residual tongue was enough to allow a complete restoration of swallowing function, with an easier, lighter, faster, and single-step procedure. In the case of palatal defects, the main eligibility criterion is the preservation of the palatal bone, and in the case of bony defects, the matrix is contraindicated because without a preserved bed, it could not provide separation between the nose/sinus and oral cavity, with resulting failure and oronasal communication [14]. On the contrary, if the bone is fully preserved, the dermal matrix is the ideal reconstructive option, even if the periosteum is not preserved. These defects were classically approached with secondary healing and dressing, but were related to severe pain and patient discomfort, especially during the feeding process. The matrix results are really satisfactory, with the absence of all these biases related to secondary healing. However, special care should be taken in the fixation of the matrix, which could be difficult in this area, where gravity also plays against the adherence of the device to the receiving bed. In such cases, in addition to a lateral fixation to the residual mucosa or to the teeth, the preparation of an orthodontic plate that pushes the membrane over the palate and protects it during feeding and swallowing is the key point to ensure the success of the procedure and patient comfort. Dental impressions could be prepared preoperatively in order to put the plate in place immediately after surgery. If the plate is available, a nasogastric tube is still indicated for feeding, but clear liquids could also be administered on the day after surgery, further improving patient comfort. Cheek defects are another important indication for reconstruction with dermal matrix: in these cases, a direct suture is usually contraindicated because it would lead to poor results in terms of mouth opening and oral commissure distortion, the main donor site for buccinator flaps is often unavailable, and the main option is often represented by the advancement of buccal fat pad [15]. However, the amount of fat provided is not always enough for adequate reconstruction and, especially in superficial defects, when the buccinator muscle is preserved, the dermal matrix represents an excellent option for the reconstruction. In this site, the matrix suture is easier in comparison to the palate and tongue, and the use of transfixed stitches is ideal to ensure a perfect adherence to the receiving bed. Furthermore, when the matrix is fixed intraoperatively with forced complete mouth opening, during the postoperative period, the laxity of the cheek prevents accidental matrix displacement.

In general terms, what we observed in our experience with the bilayer dermal matrix is the crucial role of the silicone sheet during the healing process. In the case of accidental displacement, the results are poorer and are more similar to secondary healing. However, even if it happens after a few days, the main part of healing has already taken place; therefore, results and patient comfort will be superior to a pure secondary healing approach. We found empirically that 21 days is the ideal timing for the sheet removal, because the silicone is usually still in place, and after its removal, the underlying tissue is usually perfectly healed. It is clear that a shorter period could be adequate, but considering that the patients in our series tolerated the sheet well, we did not find any reason to further accelerate the process. On the contrary, we observed that in those with an accidental displacement earlier than 7 days, healing was impaired and more similar to a secondary healing process.

What is still unclear is the real nature of the regenerated tissue—whether it is a real mucosa, scar tissue, or subdermal tissue (as the matrix is projected to be) is still to be defined. Only studies that involve biopsy of the healed tissue could clarify this in the future, but probably this assessment is not mandatory, given that clinical results, reliability, and patient comfort are the most relevant aspects to consider in the evaluation of this particular reconstructive procedure.

### Strengths and Limitations of the Study

The present study provided an insight into the use of a dermal matrix, with a specific focus on indications, timing of sheet removal, and quality of life, which have been rarely analyzed in previous studies.

At the same time, the limitations include the relatively small number of patients enrolled (despite being in line with previous studies), absence of histological support as discussed above, and the retrospective nature of the study and its monocentric design, which limit the scientific value of the results presented.

## 5. Conclusions

The bilayer dermal matrix was demonstrated to be an excellent option for small and superficial oral cavity reconstruction if proper indications are followed. A critical role is played by the protection provided by the silicon sheet; therefore, accidental removal must be prevented with proper suture, transfixed stitches, and orthodontic supplies when feasible.

The absence of morbidity, the optimal results provided, the high satisfaction of patients in terms of postoperative comfort, the very low rate of complications, and the ease of use make these devices a useful tool in the armamentarium of oral reconstructive surgeons.

## Figures and Tables

**Table 1:** Patient clinical data extrapolated from clinical records.

Variables		Mean Value
Age		58.9
Sex	8 male 13 female	
Site tumor Surgical defect dimension N surgical treatment	16 tongue; 1 hard palate; 2 cheeks; 1 lip; 1 floor of the mouth	3.5 × 4.2 cm
	6 sentinel node biopsy 6 wait and see 9 elective neck dissection	
cTNM, pTNM (overlapped) per site	TisN0M0 3 cases Tongue 2 Palate 0 Cheek 0 Lip 1 Floor of the mouth 0 T1N0M0 12 cases Tongue 9 Palate 0 Cheek 2 Lip 0 Floor of the mouth 1 T2N0M0 6 cases Tongue 5 Palate 1 Cheek 0 Lip 0 Floor of the mouth 0	
Timing of removal of silicone sheet Complications	21 days	17.4 days
	2 bleeding	14 days

**Table 2:** University of Washington Quality of Life Questionnaire. Values are expressed as a percentage from 0 to 100, where 0 is the worst achievable result, and 100 is the best result.

Code Patient	Pain	Appearance	Activity	Recreation	Swallowing	Chewing	Speech	Shoulder	Taste	Saliva	Mood	Anxiety
**1**	100	100	100	100	100	70	100	100	100	100	100	100
**2**	75	100	75	100	75	100	100	100	100	100	100	70
**3**	100	100	100	100	100	100	100	100	100	100	100	100
**4**	100	100	70	100	75	70	100	100	100	100	100	100
**5**	100	100	100	100	100	100	100	100	100	100	100	100
**6**	100	100	100	100	100	100	100	100	100	100	100	100
**7**	70	100	100	100	100	100	100	100	100	75	100	75
**8**	70	100	100	100	100	100	100	100	100	100	70	100
**9**	100	100	50	100	70	75	100	100	100	100	100	100
**10**	100	100	100	100	100	100	100	100	100	100	100	100
**11**	100	100	100	100	75	100	75	100	100	100	100	70
**12**	100	100	100	100	100	100	100	100	100	75	100	100
**13**	100	100	100	100	100	100	100	100	100	100	100	100
**14**	100	100	75	100	70	100	75	100	100	100	100	75
**15**	100	100	100	100	100	100	100	100	100	100	100	100
**16**	100	100	100	100	100	100	100	100	100	75	100	100
**17**	100	100	100	100	100	100	100	100	100	100	100	100
**18**	100	100	100	100	100	100	100	100	100	100	100	70
**19**	100	100	100	100	100	100	100	100	100	100	100	100
**20**	100	100	100	100	100	100	100	100	100	100	100	100
**21**	100	100	100	100	100	100	100	100	100	100	100	100

## Data Availability

If users would like to have information about the study data, they can personally contact the corresponding author.
